# Structural Aspects of Decreasing the Corrosion Resistance of Zinc Coating Obtained in Baths with Al, Ni, and Pb Additives

**DOI:** 10.3390/ma13020385

**Published:** 2020-01-14

**Authors:** Henryk Kania, Mariola Saternus, Jan Kudláček

**Affiliations:** 1 Department of Advanced Materials and Technology, Faculty of Engineering Materials, Silesian University of Technology, Krasińskiego 8, 40-019 Katowice, Poland; Henryk.Kania@polsl.pl; 2 Department of Metallurgy and Recycling, Faculty of Engineering Materials, Silesian University of Technology, Krasińskiego 8, 40-019 Katowice, Poland; 3 Department of Manufacturing Technology, Czech Technical University in Prague, Technická 4, 166-07 Prague, Czech Republic; Jan.Kudlacek@fs.cvut.cz

**Keywords:** hot-dip galvanizing, zinc coatings, corrosion resistance

## Abstract

The article presented the results of tests determining the synergistic effect of Al, Ni, and Pb additions on a zinc bath on the structure and corrosion resistance of coatings obtained on low silicon steel. Analyzed coatings were produced on S235JRG2 steel with Si content of 0.02 mass%. The corrosion resistance of the coatings was compared with the corrosion resistance of the coating obtained in the "pure" zinc bath. Structure at high magnifications (SEM) was determined, as well as coating thickness and chemical composition in microspheres. The corrosion resistance of the coatings was established comparatively in standard corrosion tests in neutral salt spray and a humid atmosphere containing SO_2_. It was found that the addition of Pb to the zinc bath reduced the corrosion resistance of the coatings. In the coating structure obtained in the Zn-AlNiPb bath, lead precipitation was observed in both the outer layer and the intermediate layer of the coating. Grain boundaries were the preferred site for lead precipitation. The presence of Pb precipitates favored conditions for the creation of additional corrosion cells, which led to a decrease in the corrosion resistance of the coatings.

## 1. Introduction

The durability and reliability of steel structures during their operation in an atmospheric environment depends on the quality of corrosion protection. Zinc coatings obtained by the immersion method are the most effective and economical way to protect steel against corrosion because they provide a barrier and protective protection of the substrate [1], high resistance to mechanical loads [2], and above all, unlike paint coatings, they do not require any renovation and expensive repairs during operation.

The galvanizing of steel structures takes place in commonly used zinc baths. Currently, these are multi-component zinc baths containing various alloying additives. Such additives are introduced to the zinc bath at the same time, which will result in [3,4]:

- reduction of the oxidation intensity of the bath surface,
- limiting the effect of Si content on steel on coating growth, and
- improving the ability of liquid zinc to flow from the surface of the product when emerging from the bath.

All alloying additives to zinc bath are designed to reduce zinc consumption and improve the rational use of this metal to cover a unit surface of the product.

It is necessary to reduce the oxidation of the bath surface due to the need to reduce the amount of waste in the form of zinc ashes. For this purpose, Al is added to the bath. Its presence causes the formation of a continuous Al_2_O_3_ layer on the surface of the coating, limiting further oxidation while acting as a barrier layer [5,6]. The Al content should not exceed 0.01%. Larger additions cause that Al reacts with the chlorides contained in the flux, reducing its effectiveness and the formation of discontinuities in the coating [1].

In many cases, coatings of excessive thickness are formed. Especially, if the steel contains silicon in the Sandelin range (0.03%–0.12%) and above 0.22%, the coating growth is very fast and uncontrolled. The galvanizing of reactive steels also contributes to the transition to the bath of larger amounts of iron involved in the formation of hard zinc. Nickel is added to the zinc bath to control the excessive reactivity of the steel, especially at Si content in the Sandelin range. Its content in the bath should be between 0.04%–0.06% [7,8]. Maintaining the lower limit of Ni content in the bath enables to effectively reduce the impact of silicon on the layer growth on Sandelin steels, and maintaining the upper limit does not allow the excessive formation of hard zinc [9].

The third group of alloying elements is metals, improving the castability of liquid zinc. In this group, Pb, as well as Bi and Sn, are commonly used interchangeably. The presence of Pb in the bath has been associated in the past with the use of metallurgical zinc, containing up to 1.5% Pb for galvanizing. Lead reduces the surface tension of liquid zinc and improves the fluidity of the bath, which, in turn, enables better zinc flow from the removed product [10,11]. The zinc bath has the best fluidity with Pb content in the range of 0.4–0.5 wt.%. A higher concentration of Pb in liquid zinc reduces its fluidity [12]. However, over the years, lead has been withdrawn from the bath because of its toxicity and its harmful effects on human health and the environment. In both the EU and the US, its use as an alloying additive in a zinc bath is limited in some cases [13,14]. That is why Bi and Sn have become an alternative to the lead additive. Bismuth is not harmful to human health or the environment; moreover, the addition of 0.1 wt.% Bi gives a similar intensity of dripping zinc from the surface of the product, like about 1 wt.% Pb [15]. In addition, Bi can reduce the solubility of iron in zinc [16]. The addition of tin also improves the fluidity of zinc. Bi and Sn additives, often used together, favorably affect the quality of the coating [4]; however, with the spread of these baths, many cases of steel structure cracking as a result of the phenomenon of liquid metal embrittlement (LME) have appeared [17]. Studies in this field have shown that the cause of structure’s cracking in liquid zinc may be the presence of Bi [18] and Sn [19] in the bath. Hence, the restrictions on the amount of Sn added to 0.1 wt.% and a total Pb + 10 Bi addition of less than 1.5 wt.% [20]. LME prevention guidelines limit Bi and Sn content much more than Pb content. That is why many manufacturers, who want to maintain the proper fluidity of the zinc bath, have returned to lead and are still using lead additives, despite its harmful effects on the environment.

At present, the galvanizing process of steel structures takes place only in an alloy bath, containing various additive configurations. The zinc bath always contains Al as an additive, reducing the oxidation of the bath surface, and Ni, protecting against the effects of galvanizing the steel from Sandelin range. The third group of alloying additives, such as Pb, Bi, and Sn, which improve the fluidity of liquid zinc, are used interchangeably. Limiting the content of Bi and Sn in the bath means that the bath containing Pb is still used relatively often, and its optimal chemical composition is 0.005–0.01 wt.% Al, 0.04–0.06 wt.% Ni, and 0.4–0.5 wt.% Pb.

The purpose of their application is to achieve benefits associated with improving the economy of the galvanizing process. However, there is no information on the effect of these alloying additives on the corrosion resistance of coatings, which determines the durability and reliability of the structure during its operation.

## 2. Experimental Research

### 2.1. Materials for Research

The research aimed to determine the synergistic effect of the alloying additives, such as Al, Ni, and Pb, in the zinc bath on the corrosion resistance of coatings. The content of alloying additives was maintained at the level considered optimal. To ensure the stability of the chemical composition of the bath during experimental studies and conditions similar to real conditions, the iron was dissolved in the bath to saturation. As a reference bath, "pure" zinc was used without iron-saturated alloying elements. The chemical composition of the galvanizing baths was determined using the ARL 3460 emission spectrometer and is presented in Table 1.

Test coatings were made on 50 × 100 × 2 mm^3^ samples of steel with low silicon content S235JRG2, containing 0.02 wt.% Si. The chemical composition of the steel was determined using a Spectro Lab M8 emission spectrometer and is presented in Table 2. The galvanizing process was carried out at 450 °C, and the immersion time was 180 s. Before the galvanizing process, a standard method of surface preparation was used by acid degreasing in HydronetDase solution for 5 min, pickling in 12% HCl solution for 10 min, and fluxing in a solution of TegoFlux60 for 2 min. After fluxing, the samples were dried at 120 °C for 15 min. After the galvanizing process, the samples were cooled in water.

### 2.2. Research Scope and Methodology

In order to reveal the structure of the coatings, before corrosion tests, metallographic tests were performed on an Olympus GX51 light microscope (Tokyo, Japan) using the analySIS software, which allows image registration. The coating thickness was determined using an Elcometer 355 magnetic induction meter (Manchester, England). The final result was an average of 10 measurements on each side of the sample.

The microstructure research at high magnifications (SEM) and chemical composition in the microspheres (EDS) of the coatings were carried out using a Hitachi S-3400 N scanning microscope (Tokyo, Japan) equipped with an X-ray energy dispersion spectroscopy.

The corrosion resistance of the coatings was determined comparatively in standard corrosion tests in neutral salt spray and a humid atmosphere containing SO_2_. Neutral salt spray resistance tests were carried out in accordance with the standard PN EN ISO 9227 in the salt chamber (CORROTHERM Model 610, Erichsen, Hemer, Germany). Tests were carried out in the spray of a 5% solution of sodium chloride in distilled water at 35 °C.

To determine the unit weight changes during the test, gravimetric tests were performed after 24, 48, 96, 168, 240, 480, 720, 1000 h of sample exposure in the chamber. No corrosion products were removed from the surface of the samples. Corrosion tests in a humid atmosphere, containing SO_2_, were carried out in accordance with PN EN ISO 6988 in the Koesternich Hygrotherm chamber model 519, Erichsen (Hemer, Germany). The tests were carried out in daily cycles as exposure of the samples in the chamber for 8 h, followed by exposure in an ambient atmosphere for 16 h. The appearance of the samples was evaluated during tests every 24 h. Gravimetric tests were performed after each daily cycle during the test to determine unit changes in coating weight. In each corrosion test, 5 samples from each bath were exposed in a corrosive environment. The final gravimetric test result was, on average, 5 measurements.

## 3. Results of the Research

### 3.1. Coating Characteristics before Starting Corrosion Tests

**Figure 1:**
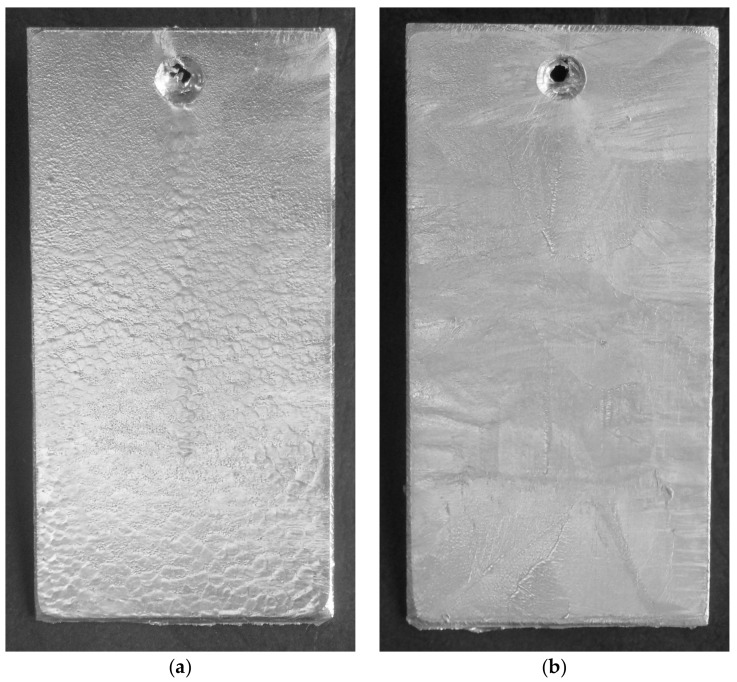
The appearance of the surface of coatings obtained in the Zn bath (**a**) and in the Zn–AlNiPb bath (**b**) before the corrosion tests.

The corrosion resistance of the coatings obtained in the Zn-AlNiPb bath was determined comparatively with respect to the coating obtained in the Zn bath. Figure 1 shows the appearance of the surface of the tested coatings. Coatings, before the corrosion tests, showed a bright and shiny appearance, which ensured the presence on the surface of the outer layer of the alloy of zinc bath. The surface of the coating obtained in the Zn bath (Figure 1a) showed the fine crystalline structure of this layer. The presence of Pb in the bath resulted in the so-called galvanizing flower with much larger grain size. The coatings did not show lumps or discontinuities.

**Figure 2:**
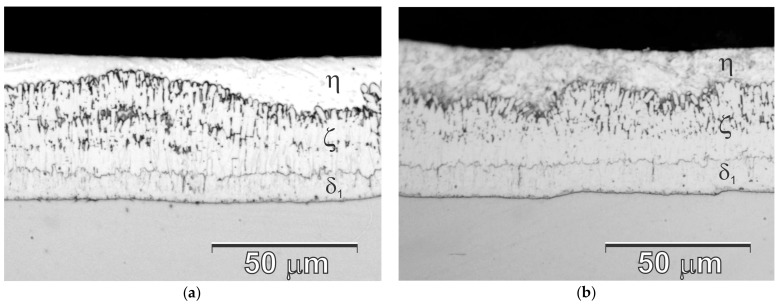
The appearance of a cross-section of coatings obtained in Zn bath (**a**) and Zn-AlNiPb (**b**) on samples for corrosion tests.

The structure of coatings obtained on samples for corrosion tests in a Zn bath and Zn-AlNiPb is shown in Figure 2. Coatings, regardless of the chemical composition of the galvanizing bath, did not show significant differences in structure. The appearance of the cross-section of the coating indicated the presence of a transition zone composed of phases of the Fe-Zn system and the outer layer η [21]. In the transition zone from the ground side, there was a phase layer δ_1_ with a compact structure and uniform thickness, which was covered with a phase layer ζ. The structure of this layer was more heterogeneous and had an uneven separation border with the outer layer of the coating. The outer layer of the coating (referred to as η—the solid solution of Fe in Zn) was actually an alloy layer of the galvanizing bath. This coating structure should be considered typical and was a characteristic of coatings obtained on low silicon steel.

**Figure 3:**
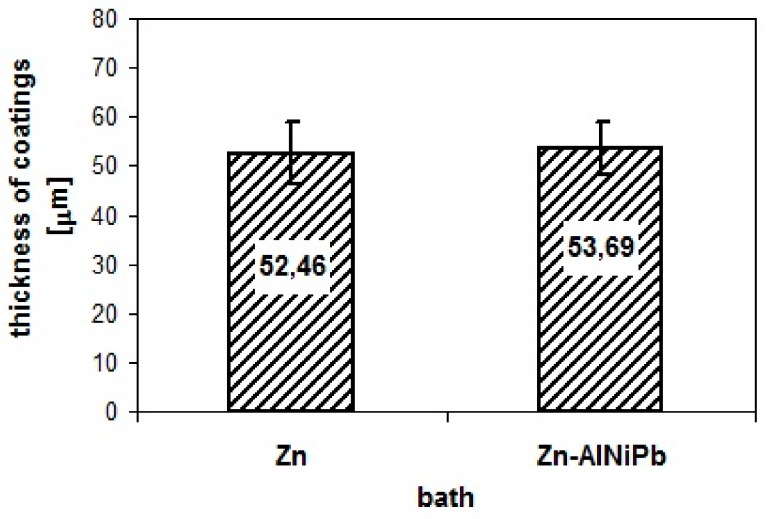
Coating thickness obtained on corrosion test samples.

Figure 3 presents the thickness of the tested coatings. The average thickness determined was 52.46 µm for the coating obtained in the Zn bath and 53.69 µm, respectively, for the coating obtained in the Zn-AlNiPb bath. Regardless of the composition of the galvanizing bath, the thickness of the coatings was very similar and met the requirements for the average and minimum value required by PN EN ISO 1461 (Warsaw, Poland). The thickness of the coating obtained in the bath containing Ni and Pb was slightly larger. It should be noted that both Ni and Pb were considered additives that reduced the thickness of the coating. Ni is responsible for reducing the thickness of Fe-Zn intermetallic layers [8,14], while Pb improves the fluidity of liquid zinc, leading to better zinc flow from the product surface and reducing the thickness of the outer layer coating [10,11]. The coating obtained in the Zn-AlNiPb bath did not, however, show such a tendency to decrease in thickness.

Based on the preliminary assessment of the structure of the coating and its average thickness, it could be concluded that the coatings obtained in the Zn and Zn-AlNiPb bath constituted a very similar material for further corrosion tests.

### 3.2. The Corrosion Resistance of Coatings in Neutral Salt Spray

**Figure 4:**
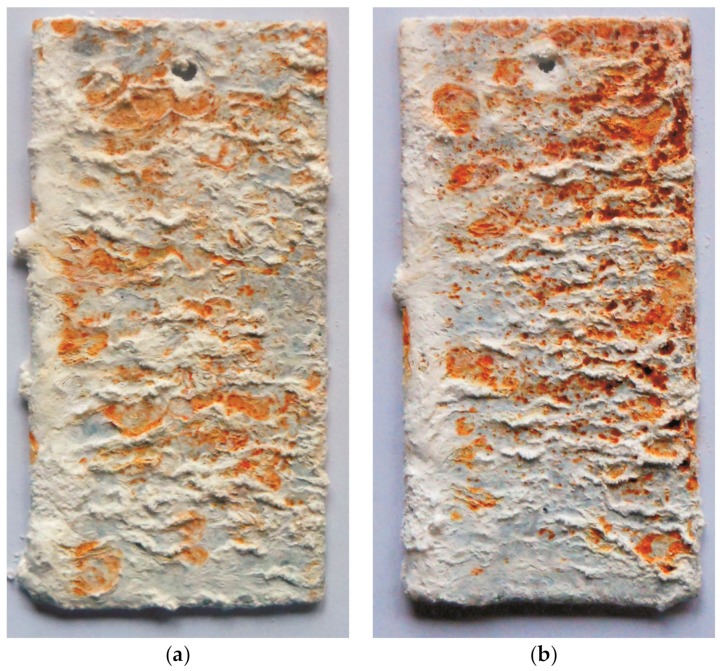
The appearance of the surface of zinc coatings obtained in the Zn bath (**a**) and in the Zn-AlNiPb bath (**b**) after 1000 h of exposure to neutral salt spray.

During the corrosion test in the salt chamber, the visual assessment of the surface of the tested coatings was carried out, as well as gravimetric measurements of mass changes. Figure 4 presents the surface appearance of coatings after 1000 h of exposure to salt spray. After completing the corrosion test, the coatings were covered with white corrosion products—whose appearance allows to state that they are zinc corrosion products [22]—and with red corrosion products—the characteristic of iron corrosion processes [23]. A much smaller proportion of red corrosion products was found on the surface of the coating obtained in the bath of "pure" zinc (Figure 4a). The color of iron corrosion products on this coating was less intense. This course of the corrosion process is characteristic of the corrosion of Fe-Zn intermetallic phases [24]. This means that after 1000 h of exposure in a salt chamber, it corroded this coating in the transition layer that had not yet lost its continuity, providing further protection. On the surface of the coating obtained in the bath containing alloying additives, it could be noticed a more intense color of red corrosion products and a clear penetration of the coating into the substrate (Figure 4b). Considering that the tested coatings had comparable thickness, the loss of continuity indicated that corrosion of the coating obtained in the Zn-AlNiPb bath progressed much faster.

**Figure 5:**
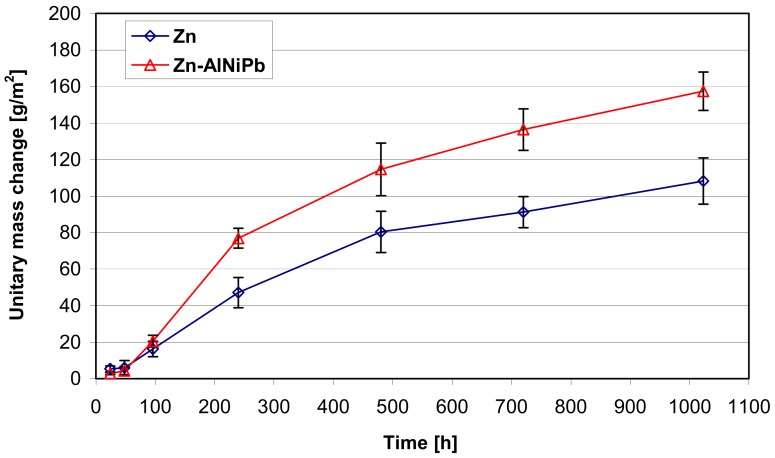
Dependence of unitary changes in the mass of coatings obtained in the Zn bath and Zn-AlNiPb during the test in neutral salt spray.

Figure 5 shows the dependence of unitary changes in coating mass during tests in neutral salt spray. Regardless of the composition of the bath, the coatings were characterized by an increase in mass during the corrosion test. The coating obtained in the bath containing alloying additions showed a greater weight gain. Based on the results obtained, it could be concluded that the coating obtained in the bath of "pure" zinc after the end of the corrosion test showed an average unit mass increase of 108.24 ± 12.66 g/m^2^. The average unit weight gain of the coating obtained in the bath containing the alloying additives Al, Ni, and Pb reached the value of 157.42 ± 10.55 g/m^2^, which gave about a 1.5-fold increase in weight compared to the coating obtained in the Zn bath. 

The available literature does not provide much information on the corrosion resistance of coatings obtained in the bath containing the alloying additives tested. Vala [25], determining the electrochemical parameters of corrosion of coatings obtained in the bath with the addition of 1%–2.5% Pb, stated that in 0.5% NaCl solution, the corrosion rate was higher compared to the corrosion rate of coatings obtained in a “pure” zinc bath. However, in a solution of 3.5% NaCl, with a content of 1 and 1.5% Pb, it showed a lower level of corrosion of coatings containing Pb.

### 3.3. The Corrosion Resistance of Coatings in a Humid Atmosphere Containing SO_2_

**Figure 6:**
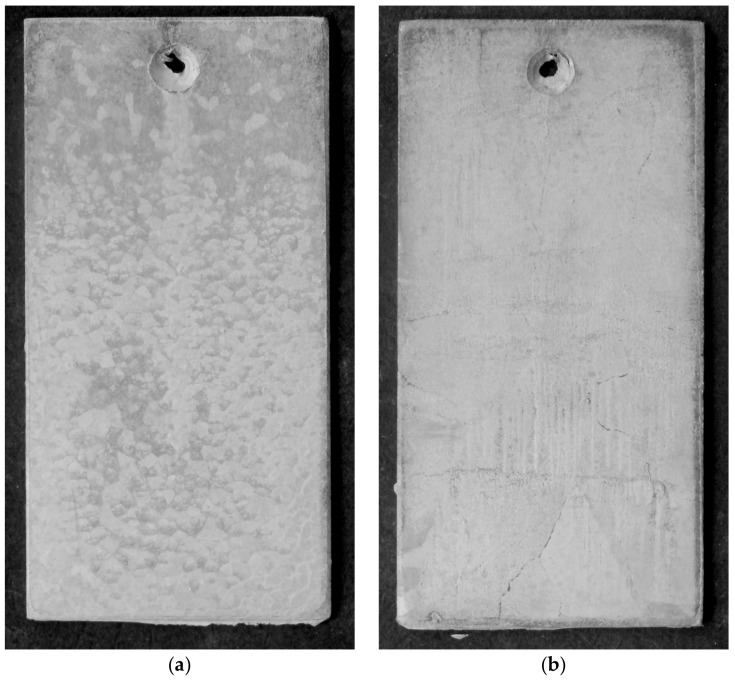
The appearance of the surface of zinc coatings obtained in the Zn bath (**a**) and Zn-AlNiPb bath (**b**) after 30 test cycles in a humid atmosphere containing SO_2_.

The appearance of the coating surface after the corrosion test in a humid atmosphere containing SO_2_ is shown in Figure 6. After 30 test cycles, it could be concluded that the coatings did not show any penetration into the substrate. Their appearance was gray and matte. The heterogeneous appearance of the surface of the coating obtained in the Zn bath, in which the occurrence of lighter and darker areas was observed (Figure 6a), indicated that the corrosion process took place in the outer layer of the coating.

**Figure 7:**
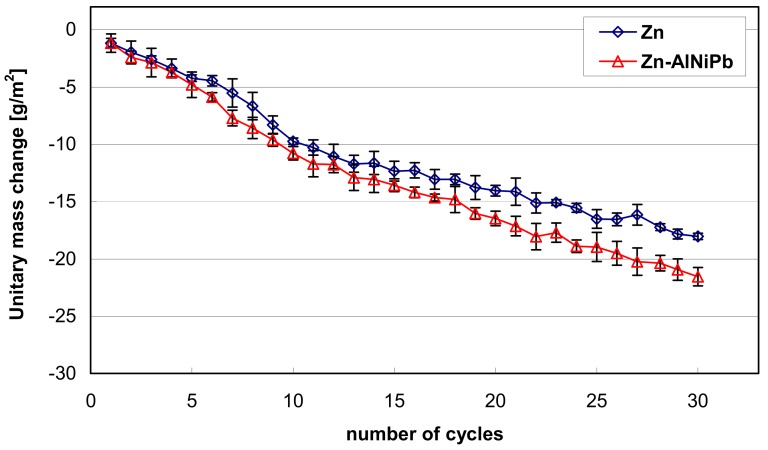
Dependence of unitary changes in the mass of coatings obtained in the Zn and Zn-AlNiPb baths during tests in a humid atmosphere containing SO_2_.

The dependence of unitary changes in coating mass during exposure in an aggressive, humid environment containing SO_2_ is shown in Figure 7. Analyzing the presented relationships, it could be concluded that the tested coatings were characterized by continuous mass loss during the corrosion test. The coating obtained in the bath, containing additions of Al, Ni, and Pb, alloy showed a greater mass loss. After the corrosion test, the unitary change in mass of this coating was reduced by 21.56 g/m^2^, while the mass loss of the coating obtained in a “pure” Zn bath was 18.04 g/m^2^. Based on the conducted corrosion test, it could be stated that the outer layer of the coating obtained in the Zn-AlNiPb bath in a humid environment containing SO_2_ underwent more intense dissolution as compared to the outer layer of the coating obtained in the "pure" Zn bath.

### 3.4. Microstructure (SEM) and Microanalysis (EDS) of the Coating Surface

**Figure 8:**
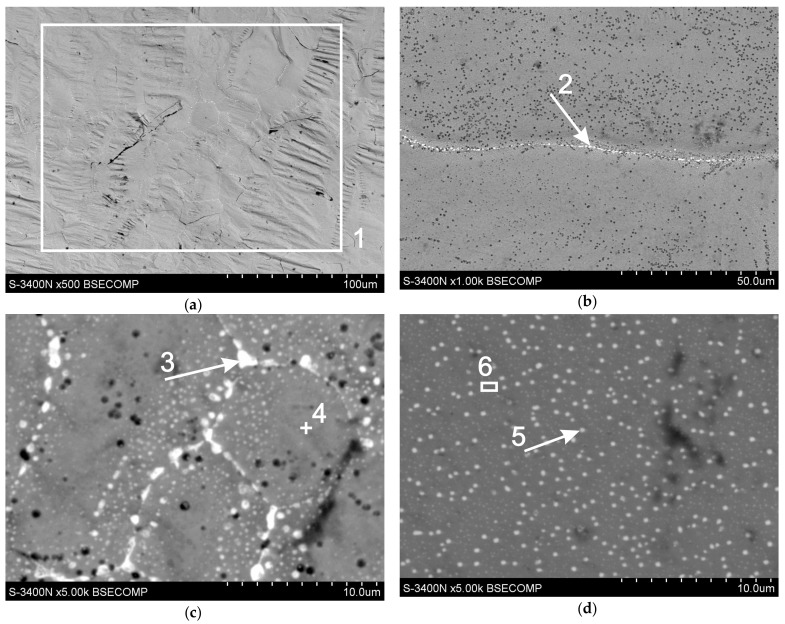
Microstructure (SEM) of the surface of the obtained coating in a Zn-AlNiPb bath: (**a**) view of the coating surface, (**b**, **c**) Pb precipitation at grain boundaries, and (**d**) Pb precipitation inside the grain.

The outer layer of the coating, which is a layer of solidified zinc bath alloy, provides a coating with a bright and shiny appearance but, at the same time, performs protective functions in the initial period of its use in a corrosive environment. Figure 8 shows the microstructure of the surface of a coating obtained in a bath containing Al, Ni, and Pb alloying additives. The chemical composition in the marked micro-areas is shown in Table 3. The microanalysis of the chemical composition carried out on the surface of the coating from an area of 0.03 mm^2^ (Figure 8a, point 1, Table 3) showed the presence of 3.5 wt.% Pb and 96.5 wt.% Fe. The determined Pb content was higher than the Pb content in the bath and significantly exceeded the limit solubility of Pb in Zn in the solid-state. According to the Zn-Pb equilibrium system, lead does not show solubility in zinc in solid state [26]. It must, therefore, be assumed that Pb is separated from the solid solution, forming precipitation in the zinc matrix. This is confirmed by studies carried out at high magnifications, in which Pb is separated from the solid solution in the form of light precipitation. Zinc grain boundaries are the preferred site for Pb segregation. Figure 8b shows that the grain boundary was continuously filled with lead.

The conducted microanalysis of the chemical composition in point 2 confirmed the presence of 39.9 wt.% Pb (Table 3). However, in Figure 8c, discontinuous Pb precipitations are visible along grain boundaries (point 3—65.5 wt.% Pb). Grain interiors could also fill small lead dispersions locally. Areas rich in finely dispersive Pb precipitations, such as those shown in Figure 8d in point 5 (Table 3, point 5—20.7 wt.% Pb), were located in the immediate vicinity of the grain boundaries. Microanalysis carried out inside the grain at point 4 indicated the presence of pure zinc in this area (Table 3, point 4). Al and Ni were not found on the surface of the zinc coating as other alloying additions to the galvanizing bath.

### 3.5. Microstructure (SEM) and Microanalysis (EDS) on the Coating Cross-Section

**Figure 9:**
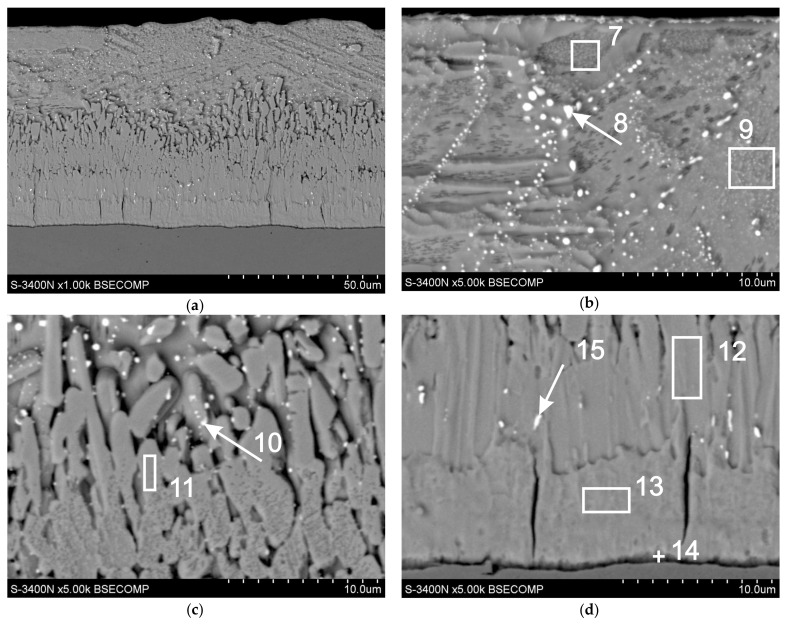
Microstructure (SEM) of the cross-section of the coating obtained in the Zn–AlNiPb bath: (**a**) view of the cross-section of the coating, (**b**) outer layer, and (**c**) and (**d**) transitional layer of the coating.

The microstructure of the coatings obtained in the Zn-AlNiPb bath is shown in Figure 9, while the percentage share of the analyzed elements is presented in Table 4. 

The microanalysis of the chemical composition allowed to state that the lead in the coating caused precipitation in the outer layer but also in the transitional layer of the coating. In the outer layer (Figure 9b), they could be individual deposits up to 1 µm, as in point 8 (Table 4, point 8—60.3 wt.% Pb), but they could also be smaller deposits with a clear orientation most likely at the grain boundaries. In addition, the cross-section of the outer layer might also show the presence of areas with fine dispersion Pb (point 9, Table 4) and areas of pure Zn (point 7, Table 4).

In the transitional layer of the coating (Figure 9c,d), the determined chemical composition was largely consistent with the content of components in the intermetallic phases of the Fe-Zn system: in points 11 and 12—phase ζ ( (5.9 and 6.3 wt.% Fe), in point 13—phase δ_1_ (12.5 wt.% Fe), and at point 14—phase Γ (22.8 wt.% Fe). At the same time, no Pb content was found in these phases, which indicated the lack of solubility of this element in the intermetallic phases of the Fe-Zn system. In the transitional layer, lead was released mainly around the area of the ζ phase, as evidenced by its high concentration in points 10 and 15. In the layer of phase ζ, the privileged place for the formation of Pb precipitation was the crystal boundaries of this phase (point 10), but Pb also separated in the compact zone near the separation boundary with phase δ_1_ (point 15). However, no Pb precipitation was observed in the δ_1_ phase layer. However, it should be noted that the δ_1_ phase increased in the solid phase, also in the solid phase Γ and ζ, which did not contain lead. Al and Ni were not found in the intermetallic Fe-Zn phases and in the cross-section of the outer coating layer, although they are components of the galvanizing bath.

## 4. Discussion of Research Results

Analyzing the current state of knowledge, it was found that there is little information on the relationship between commonly used alloying additives to the baths on the corrosion resistance of the coating. Aluminum is considered a metal that improves corrosion resistance at high content in the bath [27]. When considering zinc alloys, it should be taken into account that a small amount of aluminum additives reduces the corrosion resistance, especially if they have also a lead as a result of the alloy’s tendency to intergranular corrosion. The maximum decrease occurs at 0.3% Al in the alloy containing 0.2% Pb [28]. However, the influence of Al on the corrosion resistance of coatings with its content up to 0.01 wt.%, which is used in zinc hot-dip galvanizing methods, has not been studied. At the same time, according to the Fe-Zn-Al phase equilibrium system, the influence of Al on the structure of the coating is observed at its content in the bath above 0.14 wt.% [29].

Also, in the literature, there is no information on the impact of Ni on the corrosion resistance of coatings. Nickel affects the Fe-Zn phase morphology in coatings obtained on steels from the Sandelin range. In the Ni concentration range used for batch hot-dip galvanizing process, it does not observe the formation of Ni-containing phases in the coating [9,30].

However, the tests carried out did not show the presence of Al and Ni in the structure of the coating. Therefore, it should be assumed that with a low content of these metals in the bath, at the level considered optimal, their presence in the coating is negligible. Hence, their effect on corrosion resistance is small.

The effect of lead on the corrosion resistance of coatings is also not clearly shown in the literature. According to some sources, Pb does not affect the zinc corrosion rate [28]. Anderson [31] showed that differences in the corrosion rate of various grades of zinc, containing 0.0055, 0.049, and 0.82 wt.% Pb, during 20 years of exposure in the atmospheric environment were not significant. Similar conclusions were presented by Pryor for zinc coatings obtained in baths containing 0.57 and 0.68% Pb [32]. However, according to Sere [33], the Pb content in the bath created a privileged crystallographic orientation on the surface of the coating, which not only worsened the appearance of the coating but also reduced the corrosion resistance of galvanized steel sheets. On the other hand, Pyun [34] claimed that the addition of Pb promoted anodic dissolution of the zinc coating in an alkaline solution in all potential ranges.

The reduced corrosion resistance of the coatings obtained in the Zn-AlNiPb bath could be explained by the presence of Pb precipitations. Arenas [35] claimed that the interstitial location of lead could cause stresses in the crystallographic network, creating greater heterogeneity on the surface, thus increasing its activity. Therefore, an even corrosion process should form on the surface of the coating. However, Komorowski [36] showed greater corrosion activity in the Pb precipitation area. Electrochemical corrosion occurs when two metals differ in the electrode potential and are in the electrolyte environment. This led to the formation of corrosive cells. Thus, the disclosed Pb precipitation in the zinc matrix or Fe-Zn intermetallic phase resulted in the formation of additional corrosive cells. These cells formed already at the initial stage of corrosion in the outer layer of the coating but also at later stages during the degradation of the transition layer of the coating. The speed of the corrosion process depends on the difference between the cathode and anode metal potential in the cell. Thus, the increase in the potential difference of two metals in contact with each other in a corrosive environment increases the efficiency of the cell. Comparing the standard potentials (Table 5), it should be stated that the potential difference between Pb and Zn was much larger than between Zn and Fe. Therefore, the presence of Pb precipitation in the coating accelerated the corrosion process of the coating.

The study showed that lead, which is insoluble in zinc, segregated along the zinc grain boundaries during crystallization of the outer layer. Therefore, grain boundaries might be privileged places for accelerated corrosion. The presence of lead might, therefore, also be a critical factor in intercrystalline corrosion in the coating.

## 5. Conclusions

The tests carried out enabled to determine the synergistic effect of Al, Ni, and Pb additions to the zinc bath on the corrosion resistance of coatings. These additives did not affect the phase morphology of the coating obtained on low-silicon steel. However, the presence of Pb in the bath released this metal both in the outer layer of the coating and in the intermetallic phase layer ζ.

The corrosion resistance tests carried out indicated a decrease in the corrosion resistance of the coatings obtained in the bath, containing the combined addition of Al, Ni, and Pb. Corrosion resistance tests in neutral salt spray showed a higher proportion of iron corrosion products on the surface of the coating obtained in the Zn-AlNiPb bath compared to the coating obtained in the “pure” Zn bath. The coating obtained in this bath after 1000 h of the salt chamber exposition also showed a breakthrough in the ground. On the other hand, the coating obtained in the bath of "pure" zinc, despite being slightly thinner, maintained the coating continuity on the steel surface after the corrosion test in the salt chamber. The coating obtained in the Zn-AlNiPb bath also showed an accumulation of approximately 1.5 times more corrosion products on its surface compared to the coating obtained in the bath not containing alloying additives.

Similarly, greater corrosion wear of the coating obtained in the Zn-AlNiPb bath was observed in a corrosion test in a humid environment containing SO_2_. In this environment, more intense dissolution of the outer layer of the coating was observed, as evidenced by greater weight losses during exposure in the Koesternich chamber.

A significant number of Pb precipitations were found in the coating structure. The presence of these precipitations in the zinc matrix and in the intermetallic layer of Fe-Zn phases promoted the formation of additional corrosive cells. At the same time, Al and Ni were not found in the coating and were present in the bath as alloying additives. It could, therefore, be argued that the presence of lead in the galvanizing bath, in addition to adverse effects on the environment and human health, also contributes to the reduction of corrosion resistance of coatings, and thus to the reduction of durability and reliability of protected steel structures exposed to various corrosive environments.

## Figures and Tables

**Table 1:** Chemical composition of zinc baths.

Bath	Content [wt.%]						
	Al	Fe	Ni	Pb	Bi	Sn	Zn and Others
Zn	0.0002	0.031	0.0001	0.001	0.0003	0.0007	residue
Zn-AlNiPb	0.0048	0.029	0.049	0.48	0.0004	0.0006	residue

**Table 2:** Chemical composition of the steel.

Grade	Content [wt.%]					
	C	Si	Mn	S	P	Fe and Others
G235JRG2	0.138	0.021	0.743	0.0086	0.0088	residue

**Table 3:** Chemical composition in selected micro-areas on the surface of the coating obtained in the Zn–AlNiPb bath (analysis points according to Figure 8).

Analyzing points	Element Contents			
	Zn-K		Pb-M	
	wt%	at%	wt%	at%
point 1	96.5	98.9	3.5	1.1
point 2	60.1	82.7	39.9	17.3
point 3	34.5	62.5	65.5	37.5
point 4	100	100	-	-
point 5	79.3	92.4	20.7	7.6
point 6	99.4	99.8	0.6	0.2

**Table 4:** Chemical composition in selected micro-areas on the cross-section of the coating obtained in the Zn-AlNiPb bath (analysis points according to Figure 9).

Analysis Points	Content of Elements					
	Fe-K		Zn-K		Pb-M	
	wt. %	at. %	wt. %	at. %	wt. %	at. %
point 7	-	-	100	100	-	-
point 8	-	-	39.7	67.6	60.3	32.4
point 9	-	-	99.6	99.9	0.4	0.1
point 10	4.2	5.3	83.5	90.5	12.3	4.2
point 11	5.9	6.8	-	-	94.1	93.2
point 12	6.3	7.3	93.7	92.7	-	-
point 13	12.5	14.4	87.5	85.6	-	-
point 14	22.8	25.7	77.2	74.3	-	-
point 15	5.5	7.6	70.2	83.3	23.3	9.1

**Table 5:** Standard potentials of selected metals.

Electrode	Eo (V)
Zn/Zn^2+^	−0.76
Fe/Fe^2+^	−0.45
Pb/Pb^2+^	−0.13
